# *In Vitro* and *In Vivo* Antimalarial Activity of *Ficus thonningii* Blume (Moraceae) and *Lophira alata* Banks (Ochnaceae), Identified from the Ethnomedicine of the Nigerian Middle Belt

**DOI:** 10.1155/2014/972853

**Published:** 2014-05-14

**Authors:** M. O. Falade, D. O. Akinboye, G. O. Gbotosho, E. O. Ajaiyeoba, T. C. Happi, O. O. Abiodun, A. M. J. Oduola

**Affiliations:** ^1^Cellular Parasitology Programme, Cell Biology and Genetics Unit, Department of Zoology, University of Ibadan, Ibadan, Nigeria; ^2^Malaria Research Laboratories, Institute for Advanced Medical Research and Training, College of Medicine, University of Ibadan, Ibadan, Nigeria; ^3^Department of Public and Allied Health, Babcock University, Ilishan-Remo, Ogun State, Nigeria; ^4^Department of Pharmacology and Therapeutics, College of Medicine, University of Ibadan, Ibadan, Nigeria; ^5^Department of Pharmacognosy, Faculty of Pharmacy, University of Ibadan, Ibadan, Nigeria; ^6^University of Ibadan Research Foundation, University of Ibadan, Ibadan, Nigeria

## Abstract

Drug resistance in *Plasmodium falciparum* requires that new drugs must be developed. Plants are a potential source for drug discovery and development. Two plants that used to treat febrile illnesses in Nigeria were tested for *in vitro* and *in vivo* antimalarial activity and cytotoxicity in cancer cell lines. Methanol, hexane, and ethyl acetate leaf extracts of *Ficus thonningii* and *Lophira alata* were active in *in vitro* assays against *P. falciparum* NF54 (sensitive) and K1 (multiresistant) strains. Hexane extracts of *F. thonningii* and *L. alata* were the most effective extracts in *in vitro* assays with IC_50_ of 2.7 ± 1.6 *μ*g/mL and 2.5 ± 0.3 *μ*g/mL for NF54 and 10.4 ± 1.6 *μ*g/mL and 2.5 ± 2.1 *μ*g/mL for K1 strain. All extracts were nontoxic in cytotoxicity assays against KB human cell line with IC_50_ of over 20 *μ*g/mL, demonstrating selectivity against *P. falciparum*. *In vivo* analysis shows that hexane extracts of both plants reduced parasitaemia. At the maximum dose tested, *L. alata* had a 74.4% reduction of parasitaemia while *F. thonningii* had a reduction of 84.5%, both extracts prolonged animal survival in mice infected with *P. berghei* NK65 when compared with vehicle treated controls. The antiplasmodial activity observed justifies the use of both plants in treating febrile conditions.

## 1. Introduction

The need for the development of improved therapeutics for treatment of parasitic diseases is pressing, particularly for the treatment of malaria, where drug resistance to cheap and affordable antimalarial drugs such as chloroquine and sulfadoxine/pyrimethamine is widespread. In addition, the gradual decline in the efficacy of artemisinin-based combination therapies (ACTs) in some malaria endemic areas is a cause for concern [1]. With no effective vaccine in sight and resistance of the vector to insecticides being a problem, there is a need for the development of novel entities that would be effective against resistant parasite infections, especially* Plasmodium falciparum,* the deadliest and most common of the human malaria parasites [2]. For centuries, plants have served as a rich source of novel compounds for the treatment of various human diseases. Antimalarial drugs, developed from plants, include quinine from cinchona bark and artemisinin from* Artemisia annua*. Artemisinin is presently a key component of drug combinations introduced for treatment of resistant infections [3]. Ethnobotanical survey plays an important role in the identification, selection, and development of therapeutic agents from medicinal plants. In Nigeria and most parts of Africa, a large percent of the population depends on plant extracts, which are still widely used in the treatment of malaria and several other diseases, in particular in areas where access to standard treatments is limited. However, the potential of many of these plants as sources of antimalarial drugs has yet to be fully explored scientifically [4]. An ethnographic evaluation of ethnomedicine in the middle belt area of Nigeria revealed that many herbal remedies are used traditionally for the treatment of febrile illnesses [5]. Two of the most mentioned plants in this study were* Ficus thonningii* Blume (Moraceae) and* Lophira alata* Banks (Ochnaceae). Leaves from* F. thonningii* are used in traditional medicine in Nigeria for toothache, with added analgesic and anti-irritant properties [6]. Moreover, the leaves of* L. alata* have been known locally to treat febrile conditions, cough, jaundice, and gastrointestinal disorders [7]. This study presents the results obtained from the evaluation of the* in vitro* antiplasmodial and* in vivo* antimalarial activities of these two plants. The possible cytotoxic activities of these plants were also determined in order to determine their selective indexes.

## 2. Materials and Methods

### 2.1. Plant Collection and Authentication

Samples of the two plants were collected from various locations in Benin, Edo State, Nigeria. Mr. Usang Felix, a taxonomist at the Forestry Research Institute of Nigeria (FRIN), Ibadan, authenticated the plants, where voucher specimens were deposited under FHI numbers 107253 for* F. thonningii* and 107252 for* L. alata*.

#### 2.1.1. Plant Extraction

Plants leaves from the two plants were air-dried for approximately 48 h and ground using a simple tabletop blender. From each plant, 500 g dried leaves were extracted into redistilled methanol, hexane, and ethyl acetate by maceration at room temperature (29–33°C) for 72 h. All plant solutions were filtered and concentrated to dryness under reduced pressure using a rotary evaporator (Heidolph Laborata 4001, Germany). The extracts were stored in a refrigerator (4–12°C) until required for all assays.

### 2.2. Antimalarial Activity

#### 2.2.1. Parasites

Two* P. falciparum* strains, NF54 (sensitive to chloroquine (CQ)) and K1 (CQ resistant), kindly provided by S. Kamchonwongpaisan of BIOTEC, Thailand, were used for this study. The parasites were cultivated and maintained continuously in human erythrocytes according to previously described methods [8].

#### 2.2.2. *In Vitro* Antiplasmodial Assay

The culture medium consisted of RPMI 1640 (Gibco-BRL Laboratories, Gaithersburg, MD) supplemented with 10% heat-inactivated human type O+ serum, 25 mM NaHCO_3_, 25 mM HEPES buffer (pH 7.4), and 40 *μ*g/mL gentamicin.* In vitro* antimalarial activity was determined by the [^3^H]-hypoxanthine incorporation method [9]. All plant extracts were initially diluted in DMSO and then working solutions were prepared in complete culture media. A series of 10-fold dilutions ranging from 0.01 to 100 *μ*g/mL were prepared. Aliquots (25 *μ*L) of extracts were dispensed in 96-well microtitre plates, and 200 *μ*L of 1.5% cell suspension of parasitized erythrocytes containing 1-2% parasitemia was added to each well. Total plate content was incubated in a 3% CO_2_ incubator at 37°C. After 24 h of incubation, 25 *μ*L (0.25 *μ*Ci) of [^3^H]-hypoxanthine was added to each well. The parasite cultures were further incubated under the same conditions described above for 24 h, after which parasites were harvested onto glass filter papers (Unifilter, Packard, USA). The filters were air-dried and 20 *μ*L liquid scintillation fluid (Microscint, Packard) was added. The radioactivity on the filters was then measured using a microplate scintillation counter (Topcount, Packard, USA). The IC_50_ values of plant extract activity were obtained from dose-response curves, using nonlinear dose-response curve fitting analyses with GraphPad Prism v.3.00 software. The concentration of any substance that inhibited 50% of the parasite growth (IC_50_) was determined from the sigmoidal curve obtained by plotting the percentages of [^3^H]-hypoxanthine incorporation against drug/extract concentrations.

### 2.3. *In Vivo* Antimalarial Tests

#### 2.3.1. Animals

*In vivo* tests were performed according to the NIH guide for the care and use of laboratory animals, NIH publication (volume 25, number 28), revised 1996. All animal experiments were approved by the University of Ibadan Ethical Committee on the use of laboratory animals for research. Inbred Swiss albino mice, weighing between 20 and 22 g, were used for all experiments. Animals were obtained from the animal house of the Malaria Research Laboratories, Institute for Advance Medical Research and Training (IMRAT), University of Ibadan. The mice were housed in groups of five in plastic cages, fed with mouse cubes, and provided with water* ad libitum*.

#### 2.3.2. Parasite

Chloroquine-sensitive* P. berghei *NK65 clone obtained from the Malaria Research and Reference Reagent Resource (MR4) and maintained in mice by serial passage was used in all the* in vivo *animal studies. Parasitized red blood cells were obtained from donor-infected mouse by cardiac puncture in acid citrate dextrose (ACD) anticoagulant.

#### 2.3.3. *In Vivo* Schizontocidal Activity 4-Day Peters' Test

Peters' 4-day test was used for the* in vivo* drug tests [10]. Mice were inoculated intraperitoneally (i.p.) with 200 *μ*L of 1 × 10^7^ red blood cells infected with* P. berghei* NK65. Extract (hexane extracts were administered for* in vivo* tests because they were the most effective in* in vitro* tests) treated animals received 200 *μ*L of 100, 200, 300, 400, and 500 mg/kg body weight of extract. Two control groups of animals were used: the first group was administered chloroquine at 10 mg/kg body weight while the second group of animals received saline/10% Tween 80 (vehicle solution). Plant extracts were solubilized in vehicle solution and CQ was dissolved in distilled water. All drugs/extracts were administered 2 hours after parasite inoculation (day 0) and on days 1, 2, and 3 after infection. All extracts/drugs were given orally via a cannula. Parasitemia was determined by microscopic examination of Giemsa-stained blood films taken from tail snips on day 4 after infection and microscopically examined by counting parasitemia among 1000 red blood cells. Animal survival was monitored daily, until 30 days after infection. For all the groups of experimental mice used, survival time in days was recorded and the mean for each group was calculated. The average parasite suppression was calculated as 100 × [(*A* − *B*)/*A*], where *A* is the average parasitemia in the negative control group and *B* is the average parasitemia in the test group [11].

### 2.4. Cytotoxicity Assay

Cytotoxicity of plant extract was determined using a human cancer cell line (KB, an epidermoid carcinoma of the mouth), as formerly described [12]. The KB cells grown in standard tissue culture were diluted and added to wells of 96-well microtitre plates. Plant extracts were solubilised in DMSO and added to the cultured KB cells in microtitre plates over a concentration range of 0.03–20 *μ*g/mL and incubated for three days; negative control wells contained DMSO [13]. After three days of incubation, the cells were fixed with 100% trichloroacetic acid and stained with sulforhodamine B (SRB Fluka, cat. number 86183) [14]. The dye was solubilized with dilute Tris base and added to each well, after which absorption was measured at 515 nm with an enzyme-linked immunosorbent assay plate reader. The cell absorbance values obtained after the microtitre plates were read for each extract was averaged, from which the average value of the day zero control was subtracted and the values obtained multiplied by 100 to give a percent growth relative to the negative control incubations. The 50% inhibitory concentrations were calculated for the extracts and the positive control (ellipticine IC_50_ = 0.38 ± 0.41 *μ*g/mL), using nonlinear regression analysis.* In vitro* selectivity index was determined for each extract as the IC_50_ for KB cells/IC_50_ for* P. falciparum* NF54 [15].

### 2.5. Statistical Analysis

GraphPad Prism version 3.0 (GraphPad Software, San Diego, CA, USA) was used for all statistical analyses. Student's* t*-test was used to analyze the differences in mean parasitemia level on days following treatment initiation and analysis of variance between groups (ANOVA) was used to compare difference in percentage inhibition of parasite growth. For all statistical tests, *P* < 0.05 was considered significant.

## 3. Results

### 3.1. *In Vitro*

The* in vitro* antiplasmodial activities of the six plant extracts against the two strains of* P. falciparum*, KB cell lines, and selective indices are presented in Table 1. Of the plant extracts tested against the drug-sensitive NF54 strain of* P. falciparum*, the hexane extracts from both plants showed the best activity among the tested extracts, while the other four extracts were less active. Against the drug-resistant K1 strain, the hexane extract from* L. alata* showed the highest activity, while three (*F. thonningii* hexane and ethyl acetate extracts and* L. alata* methanol extract) showed moderate activity. The ethyl acetate extract from* L. alata* and the methanol extract from* F. thonningii* were inactive (IC_50_ > 20 *μ*g/mL) against this strain of* P. falciparum*. The plant extracts generally showed better activity against the drug sensitive NF54 strain than the drug resistant K1 strain. Overall, the hexane extracts of* L. alata* leaves were the most active of the extracts tested with IC_50_ = 2.5 ± 0.3 and 2.5 ± 2.1 *μ*g/mL against K1 and NF54, respectively. This was followed by the hexane extract of the leaves of* F. thonningii* with IC_50_ of 2.7 ± 1.6 *μ*g/mL against* P. falciparum* NF54. The IC_50 _of hexane extracts of* F. thonningii* against the K1 strain of* P. falciparum* was 10.4 ± 1.6 *μ*g/mL.

### 3.2. Cytotoxic Activities and Selective Indexes of Plant Extracts

Cytotoxic activities of* F. thonningii* and* L. alata* extracts are shown in Table 1. An extract is classified as noncytotoxic, when IC_50_ is greater than 20 *μ*g/mL [12]. The cytotoxicity results reveal that all the extracts were noncytotoxic. In this experiment, ellipticine, used as positive control drug, had an IC_50_ of 0.38 ± 0.41 *μ*g/mL. The selectivity index was also determined, which is defined as the ratio of the IC_50_ value on the cell lines to the IC_50_ value against* P. falciparum* NF54. High selectivity indices indicate a strong selective killing of the malaria parasite. All the plant extracts tested displayed selectivity for* P. falciparum* (SI > 1).

### 3.3. *In Vivo* Antiplasmodial Activity

The antimalarial activity of* F. thonningii* and* L. alata* hexane extracts against* P. berghei* NK65 strain infected Swiss albino mice using the method of 4-day suppressive test is presented in Table 2. After four days of antimalarial screening of* F. thonningii* extracts (100–500 mg/kg), chemosuppression was observed in a dose dependent manner. Mean parasitaemia (%) in the treated animals ranged from 1.2 ± 0.4 to 3.8 ± 0.4, while mean parasitaemia in the control group was 10.9 ± 1.2. Mean parasitaemia for* L. alata* extracts (100–500 mg/kg) ranged from 2.3 ± 0.4 to 6.7 ± 0.8. For all doses of hexane extracts of* F. thonningii* and* L. alata* tested, there was a significant percent inhibition of parasitaemia when compared with untreated control (*P* < 0.0001). At all doses tested,* F. thonningii* extracts were more effective than* L. alata* extracts in treating* P. berghei* infections. The average chemosuppression at the highest dose tested (500 mg/kg) was similar to that observed for the CQ treatment group.* Ficus thonningii* hexane extracts also extended the survival of animals infected, although the extracts could not provide complete protective effect, as was observed for the CQ treated group. The hexane extracts of* L. alata *also showed a dose dependent chemosuppression of parasitemia, although the chemosuppression at the highest dose was not as marked as for the* F. thonningii* extract treated group. Furthermore,* L. alata* extract was also not as potent as* F. thonningii* extract in prolonging the survival of infected animals.

## 4. Discussion

In order to deliver better drugs against resistant strains of* P. falciparum* (and recently* P. vivax*) there is a need to search for new molecules from new sources [16]. Medicinal plants have in the past been the source of some of the most successful antimalarial agents such as the quinolines and artemisinin derivatives and can still serve as such a source for drug discovery [17]. Hence, Nigeria, with a wealth of unexplored natural resources, is an ideal place to search for new drugs from plants. Previous studies on plants indigenous to Nigeria have yielded some highly active antimalarial extracts [5, 18]. In a continuance of this trend, extracts from* Ficus thonningii *(Moraceae) and* Lophira alata* (Ochnaceae) were tested in this study for* in vitro *activity against two strains of* P. falciparum *and* in vivo *against* P. berghei. *Furthermore,* in vitro* cytotoxicity activities of both plant extracts were tested against the KB cancer cell line.* In vitro *antiplasmodial tests performed with extracts of* F. thonningii *on the CQ sensitive strain* P. falciparum *NF54 revealed that the hexane extract displayed the highest activity with the methanol and ethyl acetate extracts also active, but with higher IC_50_. The hexane extracts also displayed the highest activity against the K1 strain of the parasite, albeit with lower potency than that observed for the NF54 strain. Judging from the antimalarial activities observed, it appears that the putative active principles of these extracts are present in the lipophilic part of the extract. Also, the IC_50_ values of the* F. thonningii *extracts against both parasites are comparable to those described for plant remedies of various origins that showed high antimalarial activity [19–26] indicating that* F. thonningii* leaf extracts possess some activity against* P. falciparum* and would justify the use of this plant for the traditional treatment of malaria in this part of Nigeria. A recent study of plant extracts from Congo Brazzaville [27] showed modest antimalarial activity for* F. thonningii* methanolic extract, suggesting that the extraction method is critical for obtaining the active compound(s) or that the concentrations of the active compound(s) vary depending on the locality of the plant. Previous* in vitro *antimalarial studies of other members of the Moraceae family have shown similar results to those observed for* F. thonningii* in this study. Extracts from fruits from* Artocarpus integer *(Moraceae) popular in Thailand were also found to have antimalarial activity* in vitro *[28].* Maquira coriacea *(Moraceae), a plant used by the Chacobo Indians in Bolivia for the traditional treatment of malaria, also displayed activity against* P. falciparum *[29]. The stem-bark extract of this species was active* in vitro *against the chloroquine resistant strain and inactive against the sensitive F32 strain of* P. falciparum*. Results from an* in vitro *screening of Madagascan plants used to treat malaria reported that the ethanol extracts of aerial parts of* F. reflexa *a plant representing the same genus as* F. thonningii *exhibited* in vitro *activity against the chloroquine resistant Colombian strain FcB1 [30]. Other studies, reporting on the antimalarial activity of plants used in West Africa for the treatment of malaria, found that against the FcB1 strain of* P. falciparum* ethanolic leaf extracts of* F. campensis, *another member of the genus, displayed* in vitro *antiplasmodial activity [31].

Extracts of* L. alata *leaves were also active against the different strains of* P. falciparum* tested* in vitro*. Plants of the family Ochnaceae, of which* L. alata* belongs, have not been extensively studied for their antiprotozoal activity. Previously, extracts of* L. alata *stem bark were tested against the multidrug resistant strain K1 of* P. falciparum *and were found to possess antimalarial activity [5]. The stem and leaves of* Campylospermum deltoideum *(Ochnaceae) have also been described to treat malaria and febrile illnesses in Madagascar [32]. The reports of [5] and [32] are the only ones that have been published on the use of plants from the family* Ochnaceae *for treatment of malaria. Therefore, this work represents the first report on the activities of the leaves of* L. alata *against* Plasmodium *strains. Hexane extracts from* L. alata *displayed the highest activity against the NF54 chloroquine sensitive strain of* P. falciparum in vitro*. Similar activity was also observed against the multidrug-resistant K1 strain. These results are particularly encouraging for* L. alata*, suggesting that further purification from the leaves may help isolate compounds that may be active against resistant strains of* P. falciparum*.

The* P. berghei*-infected mouse model has been widely used as a preliminary test for the* in vivo *activity of potential antimalarial agents, as it provides a preclinical indication of any* in vivo *potential bioactivity as well as possible toxicity of the sample tested [33, 34].* In vitro *systems used in screening for antimalarial activity have shortfalls, particularly as they do not take into account any prodrug effect or the role of the immune system in controlling infection [17]. For this reason, in this study the hexane extracts of both plants were also tested for* in vivo* antimalarial activity against a mouse strain* P. berghei*. Results from this study indicate that* F. thonningii *and* L. alata *hexane extracts were active against the asexual erythrocytic stages of* P. berghei* in that a dose dependent chemosuppression of parasitemia and prolongation of infected animal survival was observed in both treatment groups. Unlike in the* in vitro* tests against* P. falciparum* where* L. alata* extracts were more effective than* F. thonningii*, the inverse was observed for* in vivo* studies.* Lophira alata* extract is relatively less potent* in vivo*; this may be due to host metabolism resulting in biodegradation of the active compound(s) and/or biological differences between* P. falciparum* and* P. berghei* [33, 34].

## 5. Conclusion

The two plants tested in this study for antiplasmodial activity were selected after an ethnobotanical evaluation of phytomedicines used for the prevention and treatment of fevers in the middle belt area of Nigeria. Both plants showed activity in* in vitro* antiplasmodial and* in vivo* antimalarial assays. They also lacked cytotoxicity when tested against KB cells, justifying their use in treating febrile conditions. Detailed investigations through bioassay-guided fractionation and isolation of active compounds from these plants may lead to identification of compounds that can serve as useful leads for antimalarial drug discovery and development.

## Figures and Tables

**Table 1:** * In vitro* antiplasmodial and cytotoxicity of extracts of *F. thonningii* and *L. alata* against *P. falciparum* NF54 and K1 clones and cytotoxicity against KB cells.

Plants species	Extract	*P. falciparum* NF54 (IC_50_) *μ*g/mL	*P. falciparum* K1 (IC_50_) *μ*g/mL	KB cells (IC_50_) *μ*g/mL	Selectivity index (SI)
*F. thonningii *	MeOH	5.3 ± 2.3	21.1 ± 2.3	>20	>3.8
	Hexane	2.7 ± 1.6	10.4 ± 1.6	>20	>7.4
	Eto Ac	5.3 ± 3.1	15.3 ± 2.7	>20	>3.8

*L. alata *	MeOH	11.3 ± 3.6	5.3 ± 3.6	>20	>1.7
	Hexane	2.5 ± 0.3	2.5 ± 2.1	>20	>8.0
	Eto Ac	9.7 ± 2.7	59.4 ± 1.4	>20	>2.0

Chloroquine		0.003 ± 0.001	0.12 ± 0.036		

Artemisinin		0.0009 ± 0.0004	0.001 ± 0.0007		

Ellipticine				0.38 ± 0.41	

The IC_50_ values are expressed as mean ± SD of three different determinations per experiment of two independent experiments for antiplasmodial assays and cytotoxicity assays; SI: selectivity index = IC_50_(KB)/IC_50_(NF54); Eto Ac: ethyl acetate.

**Table 2:** *In vivo* antimalarial activity of hexane extracts of *F. thonningii* and *L. alata* against *P. berghei *NK65 infected Swiss albino mice.

Drug/extract	Dose mg/kg	Mean ± SD parasitemia (%)	Percent suppression of parasitemia	Mean survival time (days)
*F. thonningii *	100	3.8 ± 0.4	65.1	10 ± 1.0
	200	3.1 ± 0.3	70.6	12.3 ± 0.6
	300	2.1 ± 0.2	76.3	14.3 ± 1.5
	400	1.5 ± 0.3	83.4	16.6 ± 0.5
	500	1.2 ± 0.4	84.5	22.3 ± 1.5

*L. alata *	100	6.7 ± 0.8	23.6	7 ± 1.7
	200	6.0 ± 1.1	36.9	9.3 ± 1.1
	300	4.1 ± 0.6	59.7	10 ± 1.0
	400	3.0 ± 0.5	68.9	12 ± 1.3
	500	2.3 ± 0.4	74.4	15 ± 2.0

CQ		0.8 ± 0.2	89.3	>30

Saline/10% Tween 80		10.9 ± 1.2		8.3 ± 0.6

The results are expressed as mean ± SD of three determinations per experiment. Experiments were performed twice.
